# A Secure IoT–Blockchain Framework for Vaccine Cold‐Chain Monitoring With Predictive Analytics

**DOI:** 10.1002/puh2.70431

**Published:** 2026-11-01

**Authors:** Lakshmanan Muthusamy, Saravanakumar Sengottaiyan, Joshuva Arockia Dhanraj, Sriramkumar R

**Affiliations:** ^1^ Department of Computer Science and Engineering (AI&ML) Dayananda Sagar University Bengaluru South India; ^2^ Faculty of Engineering and Technology Villa College Male Maldives

**Keywords:** blockchain traceability, immunization logistics, Internet of Things (IoT), predictive analytics, secure telemetry, vaccine cold chain

## Abstract

**Objective:**

The objective of this research is to design and test a framework for secure monitoring of a vaccine cold‐chain with the help of Internet of Things (IoT) sensors, blockchain‐based provenance verification, and predictive analytics.

**Methods:**

The architecture of PharmaChain IoT Vault is an integrated system comprising low‐power IoT telemetry acquisition, secure Message Queuing Telemetry Transport (MQTT)‐based communication, integrity verification using SHA‐256, a permissioned blockchain infrastructure, off‐chain telemetry storage, and a forecasting module for transformers. A real‐world vaccine cold‐chain logistics (VCL) dataset of 26,674 observations was used for experimental evaluation, spanning 30 vaccine batches, 12 monitoring sites, and multiple distribution steps. To evaluate the feasibility of secure predictive monitoring under heterogeneous operational conditions, descriptive analysis and baseline forecasting evaluation, along with blockchain‐enabled provenance verification, were performed.

**Results:**

There was a significant temporal variability in the dataset due to the changes in storage, transportation, and distribution conditions. Forecasting experiments using a baseline autoregressive model showed that it outperformed a persistence baseline, with lower mean absolute error (12.58°C) and root mean square error (15.15°C), thereby validating the use of vaccine logistics telemetry for predictive monitoring. The blockchain layer preserved the provenance of telemetry records in a tamper‐evident manner by anchoring their SHA‐256 hashes in the ledger, enabling cryptographic integrity verification.

**Conclusions:**

It is shown that secure telemetry acquisition, blockchain‐based provenance verification, and predictive analytics can be integrated into a single framework for vaccine cold‐chain monitoring. The proposed architecture can be used to develop a system for trustworthy, intelligent management of cold chains and to illustrate the challenges of forecasting in a heterogeneous logistics environment. Future research will involve testing more complex forecasting models and testing the framework in operational conditions.

**Clinical Trial Registration:**

Not applicable. This study does not involve a clinical trial.

## Graphical abstract

This proof‐of‐concept framework integrates IoT sensing, secure MQTT/TLS communication, SHA‐256 hash verification, permissioned blockchain provenance tracking, and predictive analytics for vaccine cold‐chain monitoring. Evaluation using 26,674 logistics observations shows feasible secure telemetry management and short‐term temperature forecasting to support traceable, proactive cold‐chain oversight.

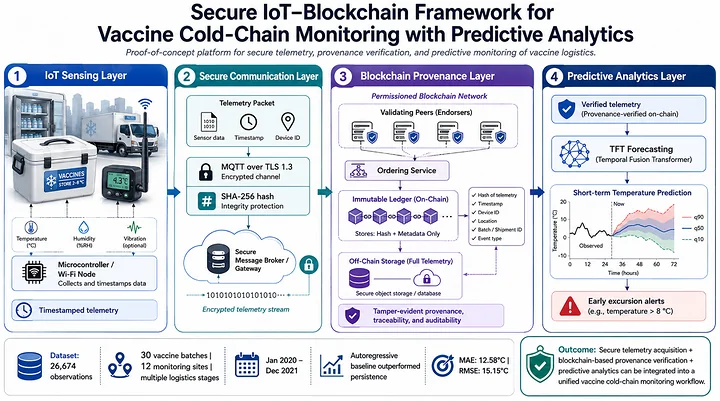

## 1 Introduction

For vaccines to be potent, the temperature must be carefully managed during storage and transport [1]. The storage of most vaccines is critical for ensuring their thermal stability, potency, and immunological activity, and they should be stored within the recommended temperature range to avoid thermal degradation and loss of potency [2, 3, 4]. Although there have been significant advances in cold chain infrastructure, vaccine distribution systems worldwide still experience temperature excursions due to equipment failures, transportation delays, poor temperature monitoring, and a lack of traceability [5, 6, 7]. These failures could lead to vaccine failures, higher operation costs, and less impact on immunization programs, especially in resource‐limited settings [8, 9, 10]. These issues drive the development of secure, intelligent monitoring systems that provide continuous environmental surveillance, trustworthy data management, and proactive risk identification.

Recent developments in Internet of Things (IoT) technology have enabled the collection and transmission of real‐time environmental telemetry data from low‐power sensing devices for continuous monitoring of vaccine storage conditions [11, 12, 13]. At the same time, permissioned blockchain platforms such as Hyperledger Fabric provide mechanisms for tamper‐evident recordkeeping, provenance tracking, and decentralized trust among participating parties [14, 15]. In parallel, machine‐learning and deep‐learning methods have shown promise in predicting environmental conditions and detecting operational patterns that indicate anomalies [16, 17, 18]. These technologies are, however, often studied as stand‐alone solutions, and very little research has examined how they can be combined into a single solution for secure and predictive vaccine cold‐chain (VCL) monitoring [19, 20, 21].

Currently available cold‐chain monitoring systems are usually limited to specific use cases, such as telemetry acquisition, cloud‐based data visualization, blockchain‐based traceability, or artificial intelligence (AI)‐driven forecasting. While conventional IoT deployments make the environment more visible, they are usually based on centralized infrastructures that may be vulnerable to data tampering, unauthorized data modification, and single points of failure [22]. While blockchain‐based solutions can improve traceability and auditability, many are limited to secure logging and record management and do not incorporate predictive analytics. Likewise, forecasting methods tend to be developed separately from methods for verifying the authenticity and integrity of sensor data that inform model predictions [23, 24]. Thus, a research gap remains in integrated solutions that combine secure telemetry acquisition, provenance verification, and predictive monitoring.

In this work, a proof‐of‐concept (PoC) framework called PharmaChain IoT Vault is proposed to overcome these limitations by integrating a single VCL monitoring framework, low‐power IoT (LP IoT) telemetry acquisition, secure Message Queuing Telemetry Transport (MQTT)‐based communication, blockchain‐based vaccine provenance verification, and predictive analytics. This framework integrates environmental sensors, permissioned blockchain technology, cryptographic integrity checks, off‐chain storage of the telemetry data, and a forecasting module built on transformers to enable proactive monitoring of cold‐chain conditions. This study is not about large‐scale deployment validation but about assessing the feasibility of bringing these technologies into a secure and trustworthy monitoring workflow, for future use in healthcare logistics.

VCL operations typically involve multiple stakeholders, including manufacturers, distributors, logistics providers, healthcare facilities, and regulatory agencies. In these distributed environments, it is imperative to have a trustworthy telemetry record to ensure regulatory compliance, support audits, and facilitate incident investigations. Centralized monitoring systems can suffer from incomplete logging, time lag in reporting, and log tampering. The proposed framework thus leverages the permissioned version of blockchain to enable a tamper‐evident provenance tracking with controlled access to Hyperledger Fabric. Notably, the blockchain part is not designed to improve forecasting results but to increase the traceability, integrity, and auditability of telemetry data. Forecasting functions work independently on verified telemetry data on blockchain to enable predictive monitoring and decision support.

This work's main contributions are outlined below:

1. Design and development of an integrated IoT–blockchain architecture for secure cold chain monitoring and telemetry management for vaccines.
2. Designing a provenance‐preservation mechanism by using a permissioned blockchain and cryptographic hash verification.
3. Including capabilities to use predictive analytics in the secure management framework for the telemetry, for proactive monitoring.
4. Experimental Evaluation – Feasibility of secure predictive monitoring with real‐world VCL logistics dataset under heterogeneous operating conditions.

This study's findings provide a foundation for future work on a unified platform for trustworthy, scalable and intelligent VCL monitoring systems that can integrate telemetry acquisition, provenance verification, and predictive analytics.

### 1.1 Related Work

#### 1.1.1 IoT VCL Monitoring

The first VCL monitoring systems were mainly based on a network of sensors, connected via the IoT with cloud‐based telemetry platforms for real‐time environmental monitoring. These systems enabled tracking of temperatures in storage areas and recording of humidity levels during vaccine storage and transport. Multiple research projects have shown that wireless sensor networks and cloud‐based monitoring systems can help detect temperature violations and reduce the need for manual oversight [25, 26]. Many IoT‐based systems rely on centralized cloud infrastructures, which may raise concerns about data integrity, auditability, and single points of failure in distributed healthcare environments [27]. Some implementations have integrated secure communication methods and incorporated cloud‐based analytics, but telemetry records are often tampered with, and insider threats can compromise the entire audit trail. Thus, the integrity and trustworthiness of environmental telemetry pose a major challenge in VCL monitoring applications.

#### 1.1.2 Blockchain‐Based Traceability and Provenance Management

Potential applications of blockchain technologies in the pharmaceutical and healthcare supply chain include enhanced traceability and tamper‐evident records management [28]. Public blockchain platforms offer immutability and decentralized consensus but can have latency issues and scalability constraints, which may not be suitable for near‐real‐time telemetry workloads. For this reason, permissioned blockchain platforms, such as Hyperledger Fabric, have gained interest for their ability to provide controlled access for organizations, support endorsement policies, and enable efficient transaction processing [29, 30]. The current blockchain‐enabled cold‐chain systems mostly target secure logging, chain of custody, and supply chain traceability. However, many implementations do not include predictive monitoring or forecasting and are therefore only able to perform retrospective analysis of event recording [31].

#### 1.1.3 Artificial Intelligence for Cold‐Chain Forecasting

In cold‐chain monitoring, machine‐learning and deep‐learning solutions have increasingly been used for temperature forecasting and anomaly detection. Conventional statistical models such as AutoRegressive Integrated Moving Average (ARIMA) and Holt–Winters techniques, while useful for interpretable forecasting, can be limited in their ability to model long‐range temporal relationships and nonlinear dynamics in cold‐chain temperatures [19]. Recurrent neural network (RNN) models such as long short‐term memory (LSTM) and gated recurrent unit (GRU) have demonstrated greater efficiencies in temporal sequence modeling but have shown some drawbacks in modeling long‐term forecasting horizons and complex multivariate dependencies [21]. More recently, transformer‐based forecasting architectures have shown strong performance on time‐series prediction tasks by leveraging attention mechanisms to capture long‐range temporal dependencies. The Temporal Fusion Transformer (TFT) offers interpretable variable selection and probabilistic forecasting capabilities, which may be useful for risk‐sensitive applications such as VCL monitoring [31]. However, a thorough comparison of transformer‐based models with statistical and RNN models remains an active area of research.

#### 1.1.4 Comparative Analysis of Existing Approaches

The existing approaches are compared in this section. The current research on cold chain monitoring mostly follows three trajectories: IoT telemetry, blockchain traceability, and AI forecasting. While IoT‐based systems provide a better sense of the environment, they are typically centralized, making them susceptible to data tampering and a lack of full auditability. Blockchain solutions offer tamper‐evident provenance tracking and decentralized record management, but many implementations are largely limited to secure logging and do not incorporate predictive analytics. Likewise, AI‐driven forecasting methods typically rely on the assumption that the telemetry data fed in are accurate and trustworthy, without specifically considering data provenance or verifying data integrity. Table 1 shows the comparison of cold‐chain monitoring systems existing on the basis of blockchain and AI.

**TABLE 1:** Comparison of cold‐chain monitoring systems existing based on blockchain and AI.

Prior work	IoT monitoring	Secure telemetry	Blockchain/ledger	AI forecasting	Probabilistic output	Key limitation
Basic cold‐chain IoT monitoring [32]	Provided	Basic encryption only	Not incorporated	Not incorporated	Not reported	Limited traceability and threshold‐based monitoring only
IoT with cloud backend [33]	Provided	Secure communication available	Centralized cloud storage	Classical ML models	Not reported	Vulnerable to centralized data management and limited auditability
Public blockchain for pharma tracking [34]	Provided	Secure transmission available	Public blockchain	Not incorporated	Not reported	High transaction latency and scalability constraints
Hyperledger‐based pharma traceability [35]	Provided	Secure communication available	Hyperledger Fabric	Not incorporated	Not reported	Primarily focused on provenance tracking without predictive analytics
IoT + blockchain for temperature logging [36]	Provided	Secure telemetry available	Hyperledger Fabric	Not incorporated	Not reported	Supports secure logging but has limited forecasting capability
AI‐based cold‐chain anomaly detection [37]	Provided	Secure communication available	Limited or absent	Classical ML methods	Not explicitly addressed	Limited support for multi‐horizon forecasting and telemetry provenance
Transformer‐based temperature forecasting [38]	Provided	Secure communication available	Not incorporated	Transformer‐based forecasting	Probabilistic estimates provided	Blockchain‐based provenance tracking has not been reported
Proposed work	Multi‐sensor low‐power IoT monitoring	TLS 1.3 and SHA‐256 hashing	Hyperledger Fabric (permissioned)	Temporal Fusion Transformer (TFT)	Quantile‐based probabilistic forecasting	Proof‐of‐concept integration of secure telemetry, blockchain provenance tracking, and predictive forecasting

#### 1.1.5 The Motivation Is to Determine the Research Gap

Although there is significant research on IoT monitoring, blockchain traceability and AI forecasting, the overall research on these technologies remains fragmented across disjointed areas. IoT‐centric systems increase environmental visibility, but they typically rely on centralized infrastructure with limited capabilities to verify resource provenance. Blockchain‐enabled solutions provide greater traceability and auditability but are primarily concerned with secure recordkeeping rather than predictive monitoring. Likewise, forecasting studies often take data integrity and data provenance verification as a given, since they rely on reliable input telemetry.

A holistic system that encompasses secure telemetry data acquisition, blockchain for provenance preservation, and predictive analytics within a single cold‐chain monitoring process remains underexplored. This study proposes PharmaChain IoT Vault, a PoC system that brings together LP IoT telemetry acquisition, secure communication, blockchain‐based provenance verification, and predictive analytics into a single VCC monitoring system. The goal of the study is to assess the viability of integrating the technologies into a secure and trustworthy monitoring workflow, not to define large‐scale deployment readiness. While the proposed framework integrates several technologies into a single architecture, the current work is a feasibility assessment to support further research on secure and intelligent VCL monitoring systems.

## 2 System Architecture

PharmaChain IoT Vault is a PoC architecture for a platform that combines IoT‐based telemetry acquisition, secure communication, blockchain‐based tracking of VCL provenance, and AI‐driven forecasting of vaccine temperatures. The framework enables the continuous environmental sensing, secure telemetry transmission, tamper‐evident ledger recording, and short‐term forecasting in a single experiment. Figure 1 provides a high‐level overview of the elements and their interrelationships within the integrated pipeline.

**FIGURE 1:**
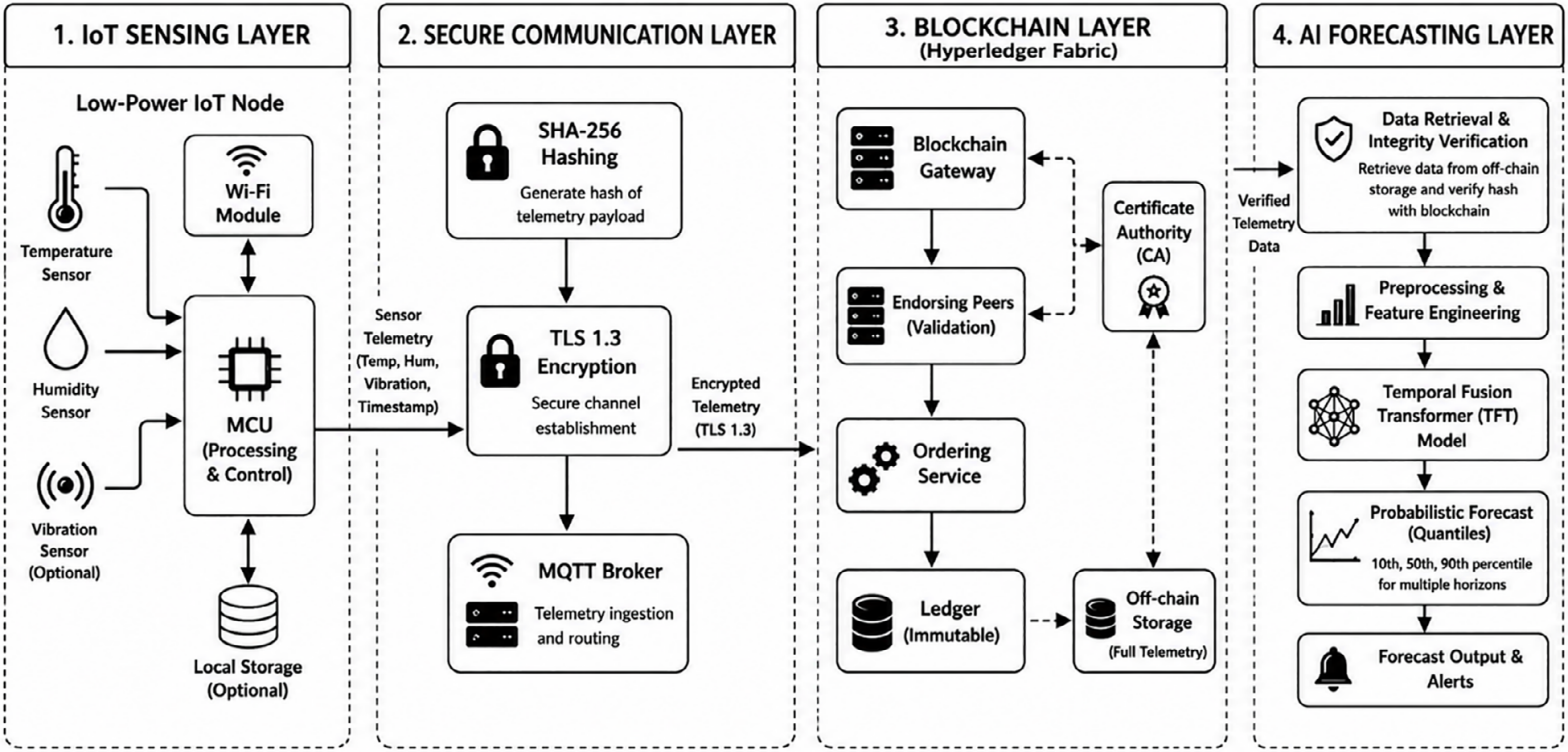
Overall system architecture of PharmaChain IoT Vault.

The general structure of the proposed PharmaChain IoT Vault framework is shown in Figure 1, which consists of four main layers: the IoT sensing layer, the secure communication layer, the blockchain provenance layer, and the AI forecasting layer. Low‐power sensor nodes are deployed in vaccine storage units to collect environmental telemetry data from the IoT sensing layer continuously. The telemetry data are securely sent using MQTT over TLS 1.3 and integrity verified using a security hash function (SHA‐256). A permissioned Hyperledger Fabric blockchain network is used to validate and add telemetry metadata to the blockchain, providing tamper‐evident provenance tracking and integration with off‐chain storage. The verified telemetry records are then analyzed by the forecasting module called TFT to produce short‐term probabilistic temperature forecasts in VCL settings. The architecture is intended as a framework for the proof of concept to test the integration of secure telemetry and the viability of predictive monitoring and is not designed for large‐scale operational deployment.

### 2.1 IoT Sensing Layer

The IoT sensing layer consists of low‐power sensor nodes that continuously monitor refrigeration conditions in vaccine storage environments. Every sensing node is composed of temperature and humidity sensors, a microcontroller unit (MCU) that acquires telemetry signals, generates timestamps, performs local preprocessing, and enables wireless communication. Sensor measurements are taken at specific time intervals and assembled into structured telemetry messages before being sent to the backend infrastructure. The measurement process in terms of the sensors is characterized as follows (Equation 1):

(1) $$\;y_{t}=T_{t}+\varepsilon_{t},$$

where $y_{t}$ denotes the reading at the time t, $T_{t}$ is the true temperature, and $\varepsilon_{t}$ represents zero‐mean sensor noise. This allows upstream forecasting models to account for sensor inaccuracy and uncertainty. The proposed sensing node architecture incorporates temperature and humidity sensing components, an MCU, wireless communication capability, optional local storage, and power management functionality. The sensing node is designed to support telemetry acquisition, timestamp generation, lightweight preprocessing, and secure communication within the proposed monitoring framework.

Figure 2 presents the hardware architecture of the IoT sensing node used to acquire environmental telemetry within the proposed framework. The sensing node integrates calibrated temperature and humidity sensors, an embedded MCU, a Wi‐Fi communication module, optional local storage, and a power management subsystem. The MCU performs telemetry acquisition, timestamp generation, lightweight preprocessing, and wireless communication via MQTT over TLS 1.3. The sensing node employs duty‐cycled sensing and communication techniques to reduce energy consumption during inactivity, enabling long‐duration operation in VCL applications. The sensors were calibrated using a laboratory reference thermometer prior to deployment in the experiment.

**FIGURE 2:**
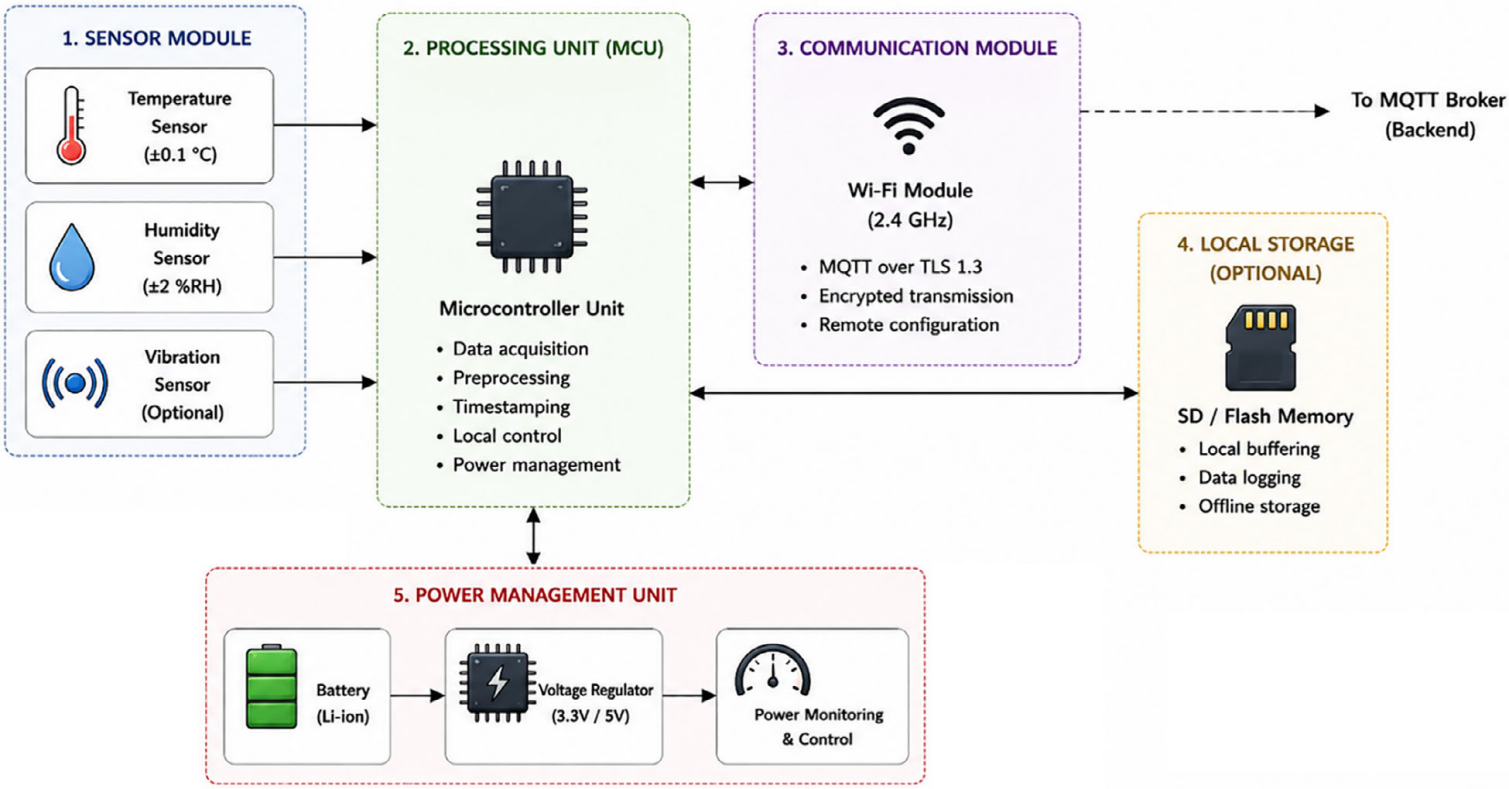
Hardware architecture of the IoT sensing node.

### 2.2 Secure Telemetry and Communication Layer

The IoT sensing layer uses the MQTT protocol with TLS 1.3 to send telemetry messages to the back‐end infrastructure. Each telemetry payload is cryptographically processed with the SHA‐256 hash function before transmission, creating a “fingerprint” (a hash) that is related to the sensor record in a tamper‐evident way. Later, this hash is used to anchor it in the blockchain ledger, aiding integrity checks and provenance tracking. Prior to transmission, each telemetry payload p_t_ is hashed using SHA‐256 to obtain its cryptographic fingerprint h_t_, as given in (Equation 2):

(2) $$\;h_{t}=\mathrm{SHA}256\left(p_{t}\right),$$

where $p_{t}$ is the raw payload data at time *t*. This hash is kept on‐chain to link each sensor message to a verifiable entry in the on‐chain ledger. In the framework, integrity checks are performed using cryptographic hashing, and no digital signatures are used to authenticate the device. TLS certificate‐based authentication between the IoT node and the MQTT broker is used to authenticate devices and ensure encrypted communication.

Figure 3 illustrates the secure telemetry communication workflow implemented in the proposed framework. Environmental telemetry from the IoT sensing node is first processed with the SHA‐256 cryptographic hash function to produce an integrity‐preserving telemetry fingerprint. The telemetry payload and corresponding hash are then transmitted securely via MQTT over TLS 1.3, with mutual certificate‐based authentication between the IoT device and the MQTT broker. After successful ingestion by the broker, the telemetry metadata and associated hash are forwarded to the Hyperledger Fabric blockchain gateway for endorsement, ordering, and recording on the immutable ledger. This workflow enables confidential telemetry transmission, tamper‐evident provenance tracking, and secure integration between the IoT sensing infrastructure and the blockchain network. The blockchain layer stores telemetry hashes and metadata rather than complete sensor payloads to reduce ledger storage overhead while preserving the ability to verify integrity.

**FIGURE 3:**
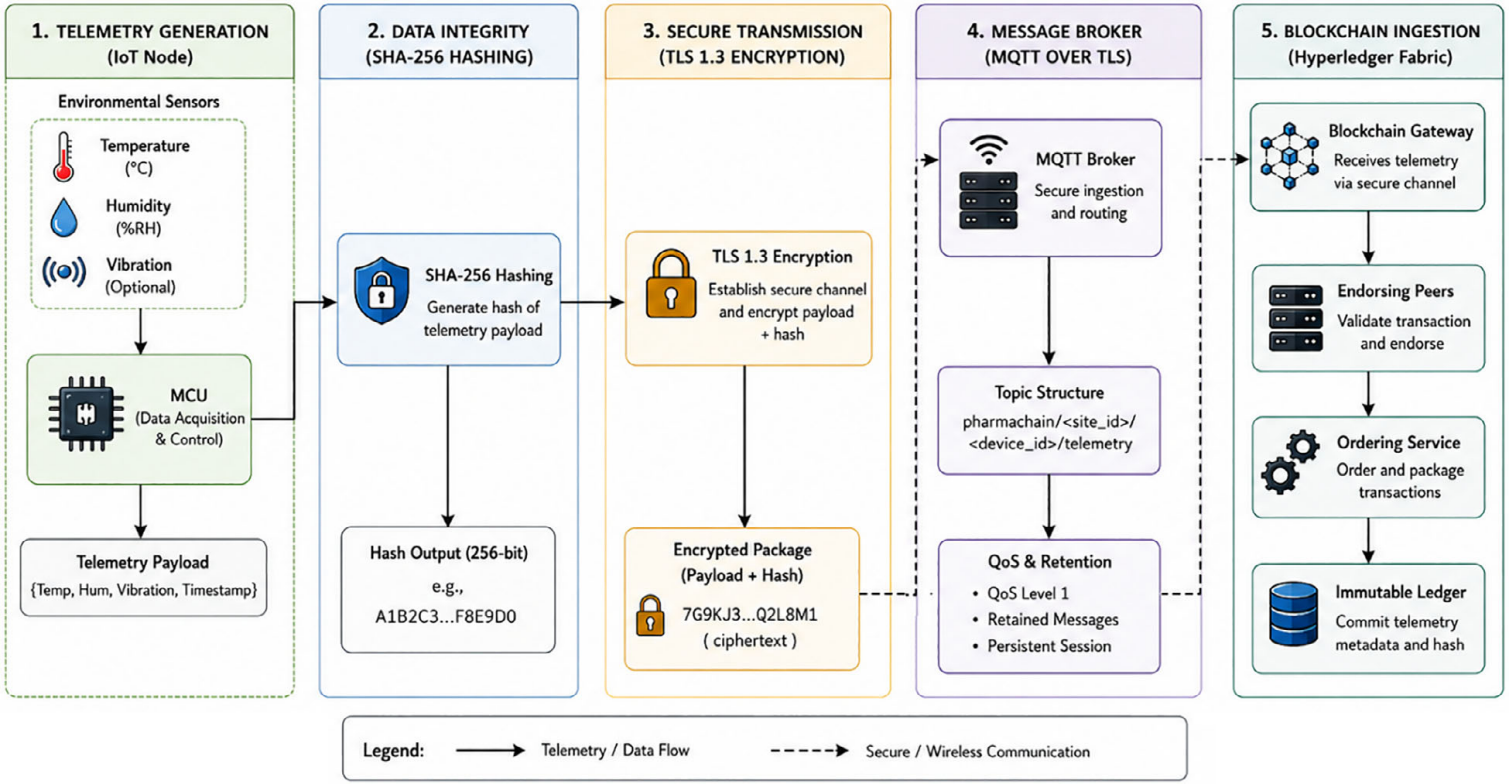
Blockchain‐based telemetry and validation process flow.

### 2.3 Blockchain‐Based Integrity and Traceability Layer

A permissioned Hyperledger Fabric blockchain network is proposed to provide tamper‐evident provenance tracking for telemetry metadata generated by the IoT sensing layer. Rather than storing complete sensor payloads on‐chain, the framework stores SHA‐256 hashes of telemetry, timestamps, and device identifiers on the ledger while keeping full telemetry records in off‐chain storage. This hybrid design reduces blockchain storage overhead while preserving verifiable integrity linkage between telemetry records and ledger entries. The blockchain component is intended to support auditability and tamper‐evident provenance management among multiple organizational stakeholders, including vaccine distributors, healthcare facilities, and regulatory authorities. The blockchain infrastructure does not directly improve forecasting accuracy; instead, it provides integrity verification for telemetry data used in downstream analytics and forecasting.

Figure 4 illustrates the blockchain transaction workflow deployed on a Hyperledger Fabric network. Once telemetry has been generated at the IoT sensing node, the node calculates a hash of the telemetry using the SHA‐256 algorithm and sends it to the blockchain gateway along with metadata about the telemetry and device identifiers. The transaction proposal is then endorsed by peers in accordance with the endorsement policies and sent to the ordering service for block sequencing and, at the same time, for ledger commitment. Once validated and ordered, the transaction metadata and transaction telemetry hash are added to the state in the immutable ledger. To minimize the overhead of storing information on the blockchain, full telemetry payloads are stored off‐chain, and only the hashes, timestamps, and metadata references for those payloads are stored on‐chain. By combining this hybrid storage architecture with tamper‐evident provenance tracking, auditability, and scalable telemetry management, the integrity verification capability of downstream monitoring and forecasting is maintained.

**FIGURE 4:**
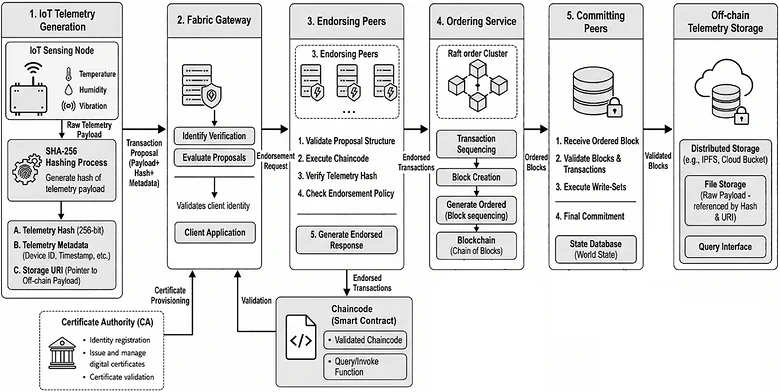
Blockchain transaction workflow.

Figure 4 shows the interaction among the components of the proposed provenance‐tracking architecture: blockchain gateway, endorsing peers, ordering service, immutable ledger, and off‐chain telemetry storage.

### 2.4 AI‐Based Forecasting Layer

The forecasting layer is based on a TFT architecture for short‐term temperature forecasting from telemetry sequences. The forecasting model receives the telemetry data from the blockchain‐verified off‐chain data storage with integrity verification. The goal of the forecasting module is to approximate future temperature fluctuations in VCL settings using multi‐horizon probabilistic forecasting (Equation 3).

(3) $$Y_{t:t+\tau }=f\left(X_{t-k:t}\right),$$

where $X_{t-k:t}$ represents the multivariate historical input window with *k* representing the forecast horizon. The forecasting layer will include a blockchain‐telemetry‐verified, multi‐horizon temperature‐forecasting component based on the TFT forecasting architecture. The forecasting framework is designed to provide prediction intervals for the lower‐bound, median, and upper‐bound forecasts, enabling uncertainty‐based monitoring and the integration of future prediction analytics.

Figure 5 shows the AI forecasting procedure using the TFT architecture. The historical data from the off‐chain telemetry are pre‐processed and verified by matching them against blockchain‐anchored hashes before being fed into the model for inference. Data cleaning, data normalization, sliding‐window sequence creation, and temporal feature extraction from multivariate refrigeration telemetry are part of the preprocessing stage. Finally, the TFT model is used to generate a multi‐horizon probabilistic temperature forecast by applying quantile‐based prediction intervals at the 10th, 50th, and 90th percentiles. Forecast outputs are used to help identify possible temperature deviations and excursions in VCL operations early on. The forecasting module is designed to assess the feasibility of short‐term predictive monitoring and *not* clinical or operational deployment readiness.

**FIGURE 5:**
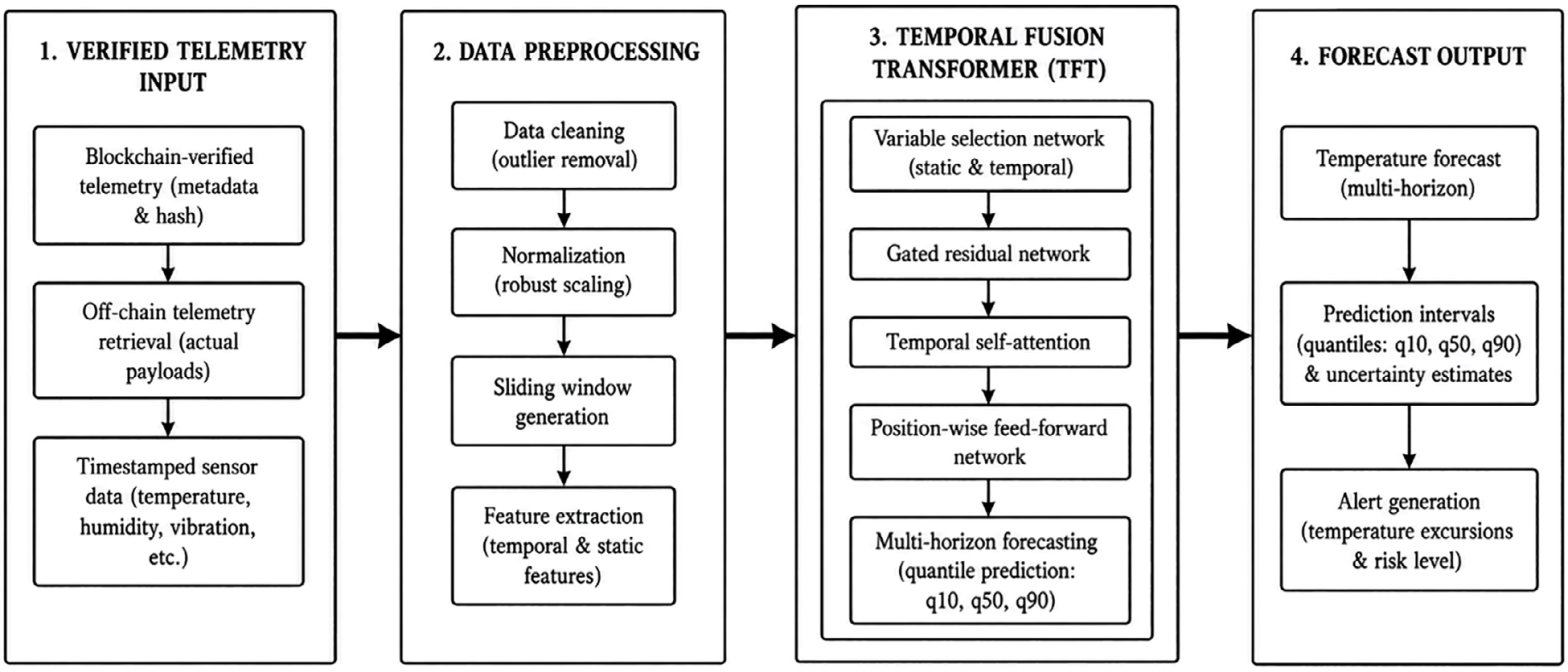
AI inference workflow.

### 2.5 The Design of the System and the Flow of Data

The different modules of the proposed framework sense, communicate, store, and forecast in an event‐driven integration workflow. IoT sensing nodes stream environmental telemetry through the secure MQTT communication layer, with SHA‐256 integrity checks, to the Hyperledger Fabric blockchain for provenance tracking and ledger commitment. Complete telemetry payloads are placed off‐chain, and hashes, timestamps, and metadata references are placed on‐chain to enable integrity verification and auditability. The telemetry data are then verified via off‐chain storage and processed by the forecasting module, the TFT, which produces short‐term probabilistic temperature forecasts. The proposed workflow illustrates interactions among telemetry acquisition, secure communication, provenance verification, off‐chain storage, and predictive analytics components within a unified architecture. The framework is intended as a PoC design for secure telemetry integration and predictive monitoring in VCL applications.

## 3 Mathematical Modeling and Forecasting

The PharmaChain IoT Vault forecasting module estimates short‐term temperature variations using blockchain‐verified telemetry from the IoT sensing infrastructure. Accurate modeling of refrigeration thermal dynamics is important because refrigeration systems may exhibit compressor‐induced oscillations, transient fluctuations, and environmental disturbances during operation. This section presents the mathematical formulation, preprocessing methodology, and probabilistic multi‐horizon forecasting strategy used in the proposed framework.

### 3.1 Forecasting Problem Formulation

The forecasting component of the proposed framework aims to estimate short‐term future temperatures based on historical multivariate telemetry sequences captured by IoT sensor networks. Let $\left(x_{t}\right)$ be the observed telemetry vector at time step (*t*), which includes temperature, humidity, temporal features derived from the timestamps, and possibly some contextual variables. The forecasting task is to predict the future temperatures for a multi‐horizon prediction window (Equation 4), given an input sequence of historical observations. $\left(X=x_{t-L+1}\;,\text{…},x_{t}\right)$ of length (L):

(4) $$Y=\left\{y_{\left\{t+1\right\}},\;\text{…},\;y_{\left\{t+H\right\}}\right\},$$

where (H) is the forecasting horizon and $\left(y_{t+h}\right)$ is the forecast for the future step (H). The observed temperature data are modelled by Equation (5):

(5) $$x_{t}=s_{t}+\varepsilon_{t}.$$

In this case, $\left(x_{t}\right)$ is the temperature measurement at time step $\left(t\right),\left(s_{t}\right)$ is the “true” temperature signal, and $\left(\varepsilon_{t}\right)$ is measurement error due to sensor uncertainty, transient environmental fluctuations or communication uncertainty. For the sake of stabilizing the training process and minimizing scale‐dependent variation, the inputs of telemetry are *z*‐score normalized (Equation 6):

(6) $$\left[z=\frac{x-\mu }{\sigma }\;\right],$$

where *μ* and *σ* are the mean and standard deviation of the training data. Temporal context features such as hour of day and day of week are encoded using cyclical sinusoidal encoding to maintain temporal periodicity (Equation 7):

(7) $$\left[\sin\left(\frac{2\pi t}{T}\right),\;\cos\left(\frac{2\pi t}{T}\right)\right],$$

where (*T*) is the temporal cycle period. A few communication failures are handled by forward‐filling or interpolation to remove breaks in the communication sequence without introducing false changes in the values. All telemetry records used for model training and inference are verified with blockchain‐anchored SHA‐256 hash values before downstream forecasting operations.

### 3.2 TFT Architecture

The forecasting framework consists of a multi‐horizon temperature forecasting model based on the TFT, a probabilistic transformer architecture. The forecasting framework is based on a probabilistic multi‐horizon temperature forecasting model with the TFT architecture. TFT models are a single class that combines recurrent sequence processing, attention‐based temporal modeling, and interpretable variable selection mechanisms. The model is equipped with gated residual networks (GRNs) for feature selection, temporal self‐attention for long‐range dependency modelling, and sequence‐to‐sequence learning for multi‐step prediction. The static contextual variables are encoded separately from the time‐dependent telemetry features, which are then combined in the temporal attention layer. The model provides a set of conditional quantiles for the lower, median, and upper bound prediction intervals, providing a probabilistic estimate of temperature behavior in a controlled monitoring environment. The TFT forecasting model forecasts the conditional distribution of future temperatures with respect to historical telemetry observations and temporal features (Equation 8):

(8) $$\left[P\left(y_{t+1:t+H}|x_{1:t},C_{t}\right)\right],$$

where $\left(x_{1:t}\right)$ is the past telemetry time series and (*C_t_*) are temporal covariates appropriate to the context. Variable selection networks (VSNs) dynamically determine the relative contribution of each telemetry feature to the forecasting process, making forecasting more interpretable and features more robust. GRNs provide stability to nonlinear transformations of features along the model pipeline, and temporal self‐attention models capture short‐term variations and long‐range patterns in the environment present in historical telemetry sequences.

### 3.3 Quantile Forecasting Objective

Probabilistic forecasting within the TFT framework is optimized using quantile regression loss. For a target observation $\left(y_{t}\right)$, predicted quantile $(\left({\hat{y}}_{t}^{\left(q\right)}\right)$, and quantile level q∈(0,1), the quantile loss is defined as follows (Equation 9):

(9) $$L_{q}\left(y_{t},{\hat{y}}_{t}^{\left(q\right)}\right)=\max\left(q\left(y_{t}-{\hat{y}}_{t}^{\left(q\right)}\right),\left(q-1\right)\left(y_{t}-{\hat{y}}_{t}^{\left(q\right)}\right)\right)$$

where asymmetric penalties are applied depending on whether the prediction underestimates or overestimates the target value. The overall forecasting objective aggregates quantile losses across all prediction horizons and quantile levels (Equation 10):

(10) $$L_{\text{total}}=\frac{1}{H|Q|}\sum_{h=1}^{H}\sum_{q\in Q}L_{q}\left(yt+h,{\hat{y}}_{t+h}^{\left(q\right)}\right),$$

where *H* is the forecasting horizon, and the set of prediction quantiles Q = 0.1,0.5,0.9 is used to train the model. To facilitate uncertainty‐aware monitoring in VCL environments, the probabilistic forecasting framework enables estimation of minimum, expected, and maximum temperature paths.

### 3.4 Discussion of the Forecasting Framework

In this study, a forecasting formulation based on predictive analytics and blockchain‐enabled cold‐chain telemetry of vaccines was proposed. The suggested forecasting module, based on a transformer model, aims to combine historical environmental observations, temporal context features, and verified telemetry data to produce probabilistic forecasts regarding the future temperature conditions. In the proposed framework, blockchain verification is a data‐integrity mechanism that ensures that forecasting models process authenticated telemetry records. The forecasting component is not tied to the blockchain system and is designed to learn relationships between environmental observations and contextual variables over time. The forecasting formulation is specifically designed to enable multi‐horizon forecasting, uncertainty estimation, and future incorporation of advanced sequence learning architectures. These capabilities are especially relevant for VCL applications, as proactive identification of potential temperature excursions can aid operational decision‐making and risk mitigation.

### 3.5 Summary and Limitations

The mathematical formulation in this section provides a framework for combining blockchain‐verified telemetry with probabilistic forecasting to monitor the VCL. The method proposed in this paper combines telemetry pre‐processing, temporal feature representation, transformer‐based forecasting, and quantile‐based uncertainty estimation within an overall analytical workflow. While the framework also includes a forecasting component that relies on a TFT model, the current research primarily focuses on architectural integration and feasibility assessment. Future work will include a detailed experimental comparison with other forecasting methods such as ARIMA, LSTM, and GRU. Further testing will be required with continuous refrigeration telemetry data and operational deployments to determine predictability, stability, and scalability.

## 4 Implementation

To support the PoC platform, the PharmaChain IoT Vault framework was implemented, combining IoT‐based telemetry acquisition, secure communication services, blockchain‐supported provenance tracking, off‐chain data storage, and transformer‐based forecasting. The goal was to determine whether these elements could be integrated into a single cold‐chain monitoring system, rather than optimizing each subsystem's performance. The sensing layer transmitted temperature data via a secure communication channel, which was verified by blockchain‐based integrity mechanisms, and the data were processed by the forecasting engine to generate a short‐term temperature prediction. The hardware architecture, secure communication workflow, the blockchain transaction process, and the forecasting pipeline used in the study are shown in Figures 2, 3, 4, 5, respectively.

### 4.1 IoT Hardware Implementation

The sensing subsystem design was conceived to capture environmental telemetry for vaccine refrigeration monitoring. The implementation comprised a temperature and humidity sensor, an embedded processing unit, wireless communication, and local telemetry handling. The sensor was observed individually, and the observation time was recorded prior to transmission. The embedded controller performed telemetry acquisition, lightweight preprocessing, packet generation, and communication management. To enable energy‐efficient operation, sensing and communication tasks were performed in a duty‐cycled fashion, with the device in an idle state between scheduled sensing activities. This reduces communication overhead and power consumption while still enabling periodic telemetry collection.

The functions of the firmware were polling the sensors, generating timestamps, formatting telemetry information, scheduling communication, and handling exceptions. Observations and telemetry data were structured before being sent to backend services. If there are intermittent communication gaps, telemetry packets could be stored locally until communication resumes. The implementation shown here was designed for testing the acquisition of secure telemetry in the context of an integrated cold‐chain monitoring scheme. Future work includes detailed Hardware optimization, Calibration characterization, and long‐term field deployment analysis.

### 4.2 Secure Communication and Gateway Services

The MQTT publish–subscribe protocol over Transport Layer Security (TLS) was used in the telemetry communication. The communication layer was designed to ensure end‐to‐end security of telemetry data transmission from sensing devices to back‐end services, with respect to integrity and confidentiality. Before transmission, each telemetry record was integrity‐preserving fingerprinted using the SHA‐256 hash function. The resulting hash was sent with the telemetry data and later used to register it on the blockchain and verify its integrity. The gateway service was responsible for receiving telemetry, verifying message integrity, temporarily storing messages, and creating blockchain transactions. Incoming telemetry data were verified, and metadata and associated hashes were sent to the blockchain subsystem. To ensure robustness with intermittent network connectivity, temporary buffering mechanisms were used. The secure communication workflow depicted in Figure 3 shows how telemetry generation, cryptographic hashing, encrypted communication, gateway validation, and blockchain registration work together. The communication layer enables the secure transfer of telemetry and is compatible with the provenance‐tracking mechanisms built into the blockchain network.

### 4.3 Blockchain Network Implementation

To test tamper‐evident telemetry provenance tracking, a permissioned blockchain design was used based on concepts from Hyperledger Fabric. The blockchain layer was composed of a gateway service, validating peers and an identity management service, an ordering component, and a distributed ledger. The gateway layer created a telemetry metadata transaction proposal for every telemetry observation, including telemetry metadata, time, and an SHA‐256 digest. Validation of transaction proposals was carried out using the predetermined endorsement strategies before sequencing and committing them to the ledger. Once the ledger commitment was made, the transaction records were made accessible for further provenance checks and audits. The blockchain solution was designed to demonstrate telemetry integrity and track provenance, not to support multiple operational stakeholders in consensus. Thus, the blockchain part can be seen as an evaluation of the integration of permissioned ledgers in VCL monitoring as a proof of concept. The transaction workflow is depicted in Figure 4, which shows the process of generating the telemetry hash, validating the proposal, ordering the transactions, committing to the ledger, and synchronizing with off‐chain storage services.

### 4.4 Off‐Chain Storage and Synchronization With Ledger

Complete telemetry payloads were stored off‐chain, and only telemetry hashes, timestamps, and metadata references were stored on‐chain to enhance storage scalability. This mixed storage technology reduces the storage required for the blockchain while maintaining its integrity verification capability. Each blockchain transaction contained a reference to the associated telemetry payload stored off‐chain. Now and then, synchronization processes checked the hashes on the blockchain with the retrieved telemetry records to ensure consistency between storage layers.

In the event of a mismatch between a stored telemetry record and its blockchain reference, the event may be marked for additional investigation. This helps to manage tamper‐evident telemetry without the storage constraint of storing full telemetry payloads on‐chain. Integration of telemetry storage with integrity anchoring can be separated to enable the system to track the provenance without compromising efficient storage utilization and retrieval performance.

### 4.5 Forecasting Module Implementation

The forecasting part employs a TFT‐based architecture to predict and monitor the blockchain using telemetry records. To ensure data integrity and provenance consistency before making the forecast, telemetry data are retrieved from off‐chain storage and verified against blockchain‐anchored hashes. Data preprocessing, temporal feature extraction, sequence generation, and probabilistic prediction are the four steps of the forecasting workflow. The framework aims to provide multi‐horizon temperature forecasts, with uncertainty intervals expressed as quantiles. These capabilities aim to enable proactive monitoring and early detection of potential temperature deviations in VCL environments. The forecasting module should be considered part of the proposed architecture and feasibility framework and should not be treated as a fully optimized, deployment‐ready forecasting solution. Future work also includes a full comparative analysis of the different forecasting models (ARIMA, LSTM, GRU, and TFT).

## 5 Experimental Evaluation

A real‐world VCL logistics dataset was used to test the proposed PharmaChain IoT Vault framework, which comprises environmental telemetry and storage information, coupled with logistics tracking records. Three aspects were evaluated: dataset characterization, forecasting feasibility, and blockchain‐based provenance verification. Given that the data cover multiple vaccine batches, sites, and distribution points, the goal was to test predictive feasibility across a variety of operational conditions, rather than optimizing forecast performance for any given refrigeration unit.

### 5.1 Dataset Description

A total of 26,674 observations were collected from 12 monitoring sites across 30 vaccine batches between January 2020 and December 2021 (Table 2) as experimental data. The dataset includes information from thermal shipper temperature measurements, ambient temperature observations, humidity measurements, storage duration indicators, and logistics tracking data related to VCL operations. This dataset was collected from a publicly available repository of VCL logistics and includes telemetry data from vaccine storage and distribution. Further, the temperature measured with the thermal shipper was chosen as the target variable for forecasting, and the environmental and operational variables as the explanatory variables. There were no major missing‐value problems to address with data imputation.

**TABLE 2:** Dataset characteristics.

Parameter	Value
Total observations	26,674
Vaccine batches	30
Monitoring locations	12
Logistics stages	10
Observation period	January 2020–December 2021
Target variable	Thermal shipper temperature
Missing observations	None identified

The dataset is a mix of vaccine‐distribution conditions—storage, transportation, and handling. This diversity provides a realistic foundation for assessing the feasibility of secure cold‐chain monitoring and forecasting.

### 5.2 The Characteristics of Data and Pre‐Processing of Data

Timestamps were first transformed into temporal features, such as month, day, and operational sequence representations, before forecasting. The numerical variables were *z*‐score‐normalized, and categorical variables (location, logistics stage, etc.) were one‐hot encoded for machine‐learning processing. Thermal shipper temperature was chosen as the primary forecasting target because it reflects cold‐chain storage conditions. Other explanatory variables included room temperature, relative humidity, time refrigerated, exposure to ultra‐low‐temperature freezers, and temperature‐excursion indicators. Table 3 shows the results of a descriptive analysis of the data, which indicate significant fluctuations in thermal shipper temperatures due to temperature changes across storage, transportation, and distribution environments that vaccines experience during vaccine logistics operations.

**TABLE 3:** Descriptive statistics of thermal shipper temperature.

Statistic	Value
Mean	−54.02°C
Standard deviation	28.55°C
Median	−67.00°C
Minimum	−71.00°C
Maximum	15.00°C

Distribution of thermal shipper temperature measurements recorded during vaccine cold‐chain operations. The histogram shows the frequency distribution of temperature measurements from thermal shippers across 30 vaccine batches recorded at 12 monitoring locations.

As shown in Figure 6, the majority of observations concern ultra‐low‐temperature storage conditions, which are essential for vaccine preservation. The number of observations is lower during transportation and handling operations, indicating the transitions in operations throughout the cold chain.

**FIGURE 6:**
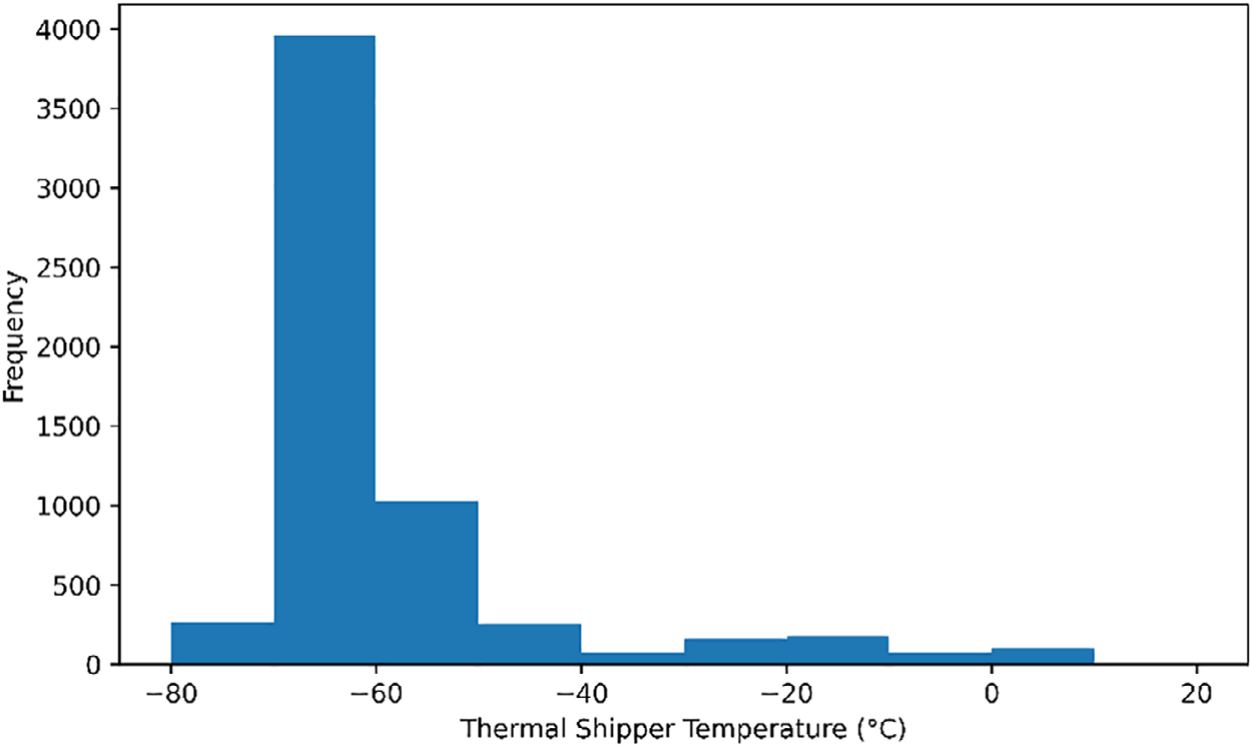
Distribution of thermal shipper temperatures.

Temporal variation in thermal shipper temperatures across the vaccine cold chain. The figure shows the temperature changes recorded for different vaccine batches and logistics phases over the monitoring period (January 2020 to December 2021). The temporal variability in the dataset is shown in Figure 7. The fluctuations observed may be attributed to the changes in the storage conditions, transportation, and handling procedures. These dynamics drive the adoption of predictive monitoring technology that detects potential temperature deviations before cold‐chain failure.

**FIGURE 7:**
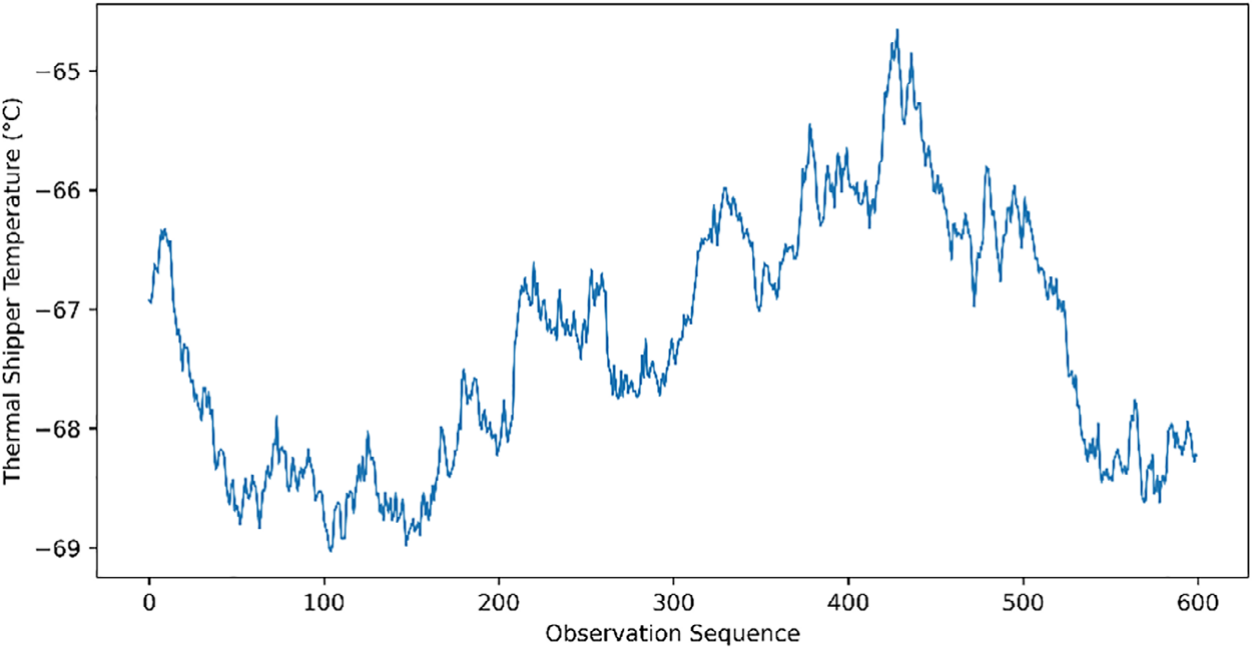
Thermal shipper temperature time series.

### 5.3 Forecasting Feasibility Analysis

To avoid information leakage, a chronological train–validation–test split was employed. About 70% of the observations were used for training, 15% for validation, and 15% for testing. Baseline time‐series models were used to assess the feasibility of forecasting. A persistence baseline was estimated by assuming that the next temperature would equal the last observed temperature. The autoregressive baseline was also examined to assess whether benefits could be obtained from simple temporal relationships. The Mean absolute error (MAE), root mean square error (RMSE), and mean absolute percentage error (MAPE) were used to measure the forecasting performance. The baseline results of forecasting performance are presented in Table 4.

**TABLE 4:** Baseline forecasting performance.

Model	MAE (°C)	RMSE (°C)	MAPE (%)
Persistence baseline	14.6	30.87	158.83
Autoregressive baseline	12.58	15.15	81.29

The autoregressive baseline outperformed the persistence baseline across all evaluation measures. The baseline evaluation aimed to assess the feasibility of forecasting using simple temporal models before exploring more complex, computationally expensive deep learning architectures. However, there was significant uncertainty in the prediction, as observations spanned various vaccine batches, storage conditions, and logistics. These heterogeneous operational conditions give rise to abrupt transitions, which are more difficult to model than continuous refrigeration telemetry streams. Error distributions revealed significant forecasting errors, especially at the change in logistics stages and storage environments. Sudden changes in the operation cause abrupt temperature changes that are hard to model with simple autoregressive models.

Actual thermal shipper temperatures for a continuous sequence of observations from a single vaccine batch, compared with the corresponding autoregressive model forecasts. Figure 8 shows the measured temperature in the thermal shipper compared with the predicted temperature for a typical vaccine batch. The autoregressive model can capture some of the temporal trend, but significant deviations are observed when the operation is undergoing rapid change, such as during storage. Single‐batch analysis allows for a more stable prediction setting than forecasting over the entire heterogeneous data, and it more closely captures the temporal dependence of the short‐term future, which is exploited by sequence‐learning methods. The findings in this paper show that forecasting in vaccine logistics settings is a difficult prediction problem that can be addressed by models capable of capturing nonlinear and temporal relationships over longer time horizons. The present forecasting analysis must therefore be considered a feasibility study rather than an optimized temperature prediction study.

**FIGURE 8:**
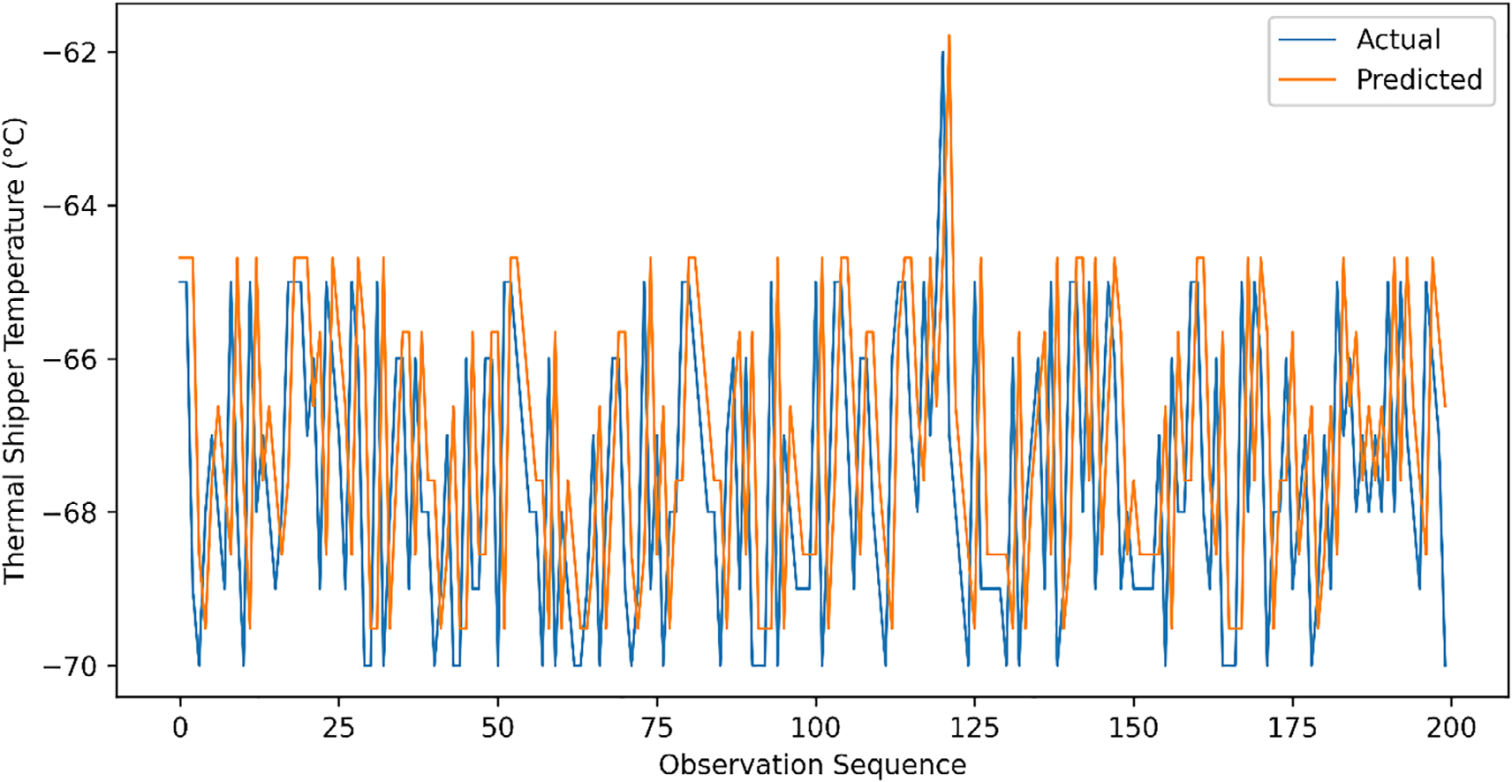
Actual versus predicted temperature values.

### 5.4 Blockchain Provenance Verification

The blockchain component was assessed from the perspectives of provenance preservation and telemetry integrity verification. An SHA‐256 hash was calculated and linked to the metadata for each observation in the telemetry before being placed on the blockchain. The hybrid storage approach involved storing entire telemetry payloads off blockchain, while storing cryptographic hashes and metadata in the permissioned blockchain ledger. This way, it was possible to verify the integrity of the telemetry without adding excessive storage overhead to the blockchain. The evaluation concluded that blockchain‐based anchoring can achieve tamper‐evident provenance tracking and that telemetry records from the cold chain can be audited. Significantly, the blockchain layer is not a forecasting enhancement mechanism but one for preserving integrity. The blockchain verification process complements the forecasting framework by ensuring that predictive models use authenticated telemetry records with independently verifiable provenance. This integration enhances trustworthiness in cold‐chain monitoring applications, where data integrity is pivotal to meeting regulatory requirements and making business decisions.

### 5.5 Discussion and Limitations

The experimental assessment demonstrates the feasibility of embedding IoT telemetry and blockchain‐based tracking of product provenance, along with predictive monitoring, within a single cold‐chain monitoring architecture. This is done with a real‐world dataset of vaccine logistics, which offers a concrete sense of the difficulties involved in forecasting in a heterogeneous setting. There are a few weaknesses to note. It is not a continuous stream of refrigeration data from a single sensor but rather several batches of data from multiple locations, spanning several logistics stages. Therefore, prediction experiments should be treated as feasibility studies rather than as optimized refrigeration‐temperature prediction studies. While the proposed architecture includes a forecasting module called TFT, only the baseline forecasting analysis was conducted with the data available for this evaluation. Preliminary forecasting evaluation was done using persistence and autoregressive models, and in‐depth comparison with ARIMA, LSTM, GRU, and TFT architectures is planned for future studies. Future studies will involve testing transformer‐based forecasting models with continuous refrigeration telemetry data, comparative forecasting experiments and testing the framework in an operational vaccine distribution environment.

## 6 Conclusion

This research proposed the PharmaChain IoT Vault, an integrated framework that combines IoT‐based telemetry acquisition, blockchain‐based provenance verification, and predictive monitoring within the VCL management system. The proposed architecture aims to address key issues in vaccine distribution, including poor traceability, a lack of verifiable environmental records, and the need for proactive monitoring of temperature‐sensitive logistics operations. The framework combines secure telemetry data collection, blockchain‐based data integrity tools, and predictive analytics into a single platform, aiming to enhance transparency and trust in VCLs. The framework includes LP IoT sensing, secure MQTT‐based communication, permissioned blockchain infrastructure, off‐chain telemetry storage, and a forecasting module that focuses on the transformer. The blockchain is designed to offer provenance verification and tamper‐evident records, and to enable auditable environmental records; the forecasting component is meant to help identify potential temperature deviations and cold‐chain risks early on.

Using a real‐world VCL logistics dataset comprising 26,674 observations across various vaccine batches, monitoring points, and distribution phases, an experimental evaluation was carried out. The results showed the feasibility of combining predictive monitoring with secure telemetry management in heterogeneous vaccine logistics environments. Baseline forecasting experiments also showed difficulties in forecasting temperature behavior when operational conditions change, especially when sudden jumps occur between storage and transportation phases. The study also provided a better understanding of how blockchain technology fits in the proposed framework. The blockchain layer does not directly improve forecasting performance but serves as a tool to preserve and verify the provenance of data, enabling traceability, auditability, and trust in cold‐chain telemetry data. This is especially relevant in vaccine supply chains, where environmental record‐keeping regulations and the need for reliable traceability are essential.

There are some drawbacks to consider. The data provided are not continuous refrigeration telemetry from a single cold storage unit but rather a collection of heterogeneous vaccine logistics operations. Additionally, the evaluation of forecasting performance was conducted only on basic temporal forecasting models, and large‐scale deployment validation was outside the scope of the present study. Hence, the results presented can be considered a feasibility study of the proposed architecture, not a full‐scale test of its operational performance. Despite these limitations, the study lays the groundwork for incorporating secure telemetry acquisition, provenance verification, and predictive analytics into future intelligent VCLs. The results demonstrate that secure telemetry acquisition, provenance verification, and prediction monitoring can be realized within a single VCL management architecture. Future studies should involve testing more sophisticated forecasting models, such as ARIMA, LSTM, GRU and TFT, using ongoing data from the refrigeration telemetry system and evaluating the framework in actual vaccine distribution settings.

## Author Contributions

**Lakshmanan Muthusamy**: conceptualization; methodology; writing – original draft; writing – review and editing. **Saravanakumar Sengottaiyan**: visualization; writing – original draft; writing – review and editing. **Joshuva Arockia Dhanraj**: methodology; hardware implementation; investigation; software development; technical review. **Sriramkumar R**: AI model development.

## Funding

The authors have nothing to report.

## Ethics Statement

This study did not involve human participants, animals, or personal/clinical data. Therefore, institutional ethical approval was not required.

## Conflicts of Interest

The authors declare no conflicts of interest.

## Data Availability

The dataset analyzed during this study was obtained from a publicly available vaccine cold‐chain logistics repository. The processed dataset and supporting analysis scripts are available at https://www.kaggle.com/datasets/manankhanna0/vaccine‐distribution‐with‐temperature‐logging, enabling independent verification and reproduction of the reported results.

## References

[1] U. Kartoglu and H. Ames , “Ensuring Quality and Integrity of Vaccines Throughout the Cold Chain: The Role of Temperature Monitoring,” Expert Review of Vaccines 21 (2022): 799–810, 10.1080/14760584.2022.2061462. 35373686

[2] N. Abbasi , N. Fnu , and S. Zeb , “AI in Healthcare: Using Cutting‐Edge Technologies to Revolutionize Vaccine Development and Distribution,” JURIHUM: Jurnal Inovasi dan Humaniora 1 (2023): 17–29.

[3] F. Goodarzian , A. Navaei , B. Ehsani , P. Ghasemi , J. Muñuzuri , et al., “Designing an Integrated Responsive‐Green‐Cold Vaccine Supply Chain Network Using Internet‐of‐Things: Artificial Intelligence‐Based Solutions,” Annals of Operations Research 328 (2023): 531–575, 10.1007/s10479-022-04713-4. PMC9071011 35540307

[4] S. Jiang , S. Jia , and H. Guo , “Internet of Things (IoT)‐Enabled Framework for a Sustainable Vaccine Cold Chain Management System,” Heliyon 10 (2024): e28910, 10.1016/j.heliyon.2024.e28910. PMC10998091 38586317

[5] V. K. Manupati , T. Schoenherr , N. Subramanian , et al., “A Multi‐Echelon Dynamic Cold Chain for Managing Vaccine Distribution,” Transportation Research Part E: Logistics and Transportation Review 156 (2021): 102542, 10.1016/j.tre.2021.102542. PMC8602632 34815731

[6] R. Raut , R. Shrestha , A. Adhikari , A. Fatima , M. Naeem , et al., “Revolutionizing Veterinary Vaccines: Overcoming Cold‐Chain Barriers Through Thermostable and Novel Delivery Technologies,” Applied Microbiology 5 (2025): 83, 10.3390/applmicrobiol5030083.

[7] S. Taseen , M. T. Yousafzai , and M. F. H. Qureshi , “Harnessing Artificial Intelligence and Digital Technology for Enhancing Routine Immunization Among Zero‐Dose Children,” Digital Health 11 (2025): 20552076251382086, 10.1177/20552076251382086. PMC12484902 41041463

[8] A. Ashok , M. Brison , and Y. LeTallec , “Improving Cold Chain Systems: Challenges and Solutions,” Vaccine 35 (2017): 2217–2223, 10.1016/j.vaccine.2016.08.045. 27670076

[9] W. Prosser , N. Danfakha , J. Makena , et al., “Cold Chain Maintenance Done Differently: Results From a Human‐Centered Design Study in Niger, Kenya and Tanzania,” Discover Health Systems 3 (2024): 97, 10.1007/s44250-024-00166-7.

[10] M. Lakshmanan , G. S. A. Mala , and R. Poorni , “Blockchain for Secure and Efficient Crowdfunding: An Optimized Particle Swarm Approach,” in 9th International Conference on Communication and Electronics Systems. ICCES 2024 (2024), 848–854, 10.1109/ICCES63552.2024.10860104.

[11] C. Sow , C. Sanou , C. Medah , et al., “Challenges of Cold Chain Quality for Routine EPI in South‐West Burkina‐Faso: An Assessment Using Automated Temperature Recording Devices,” Vaccine 36 (2018): 3747–3755, 10.1016/j.vaccine.2018.05.062. 29779921

[12] Z. Selamoglu , M. Y. Naeem , B. Selamoglu , et al., “The Use and Importance of Artificial Intelligence in Vaccine Research, Development, and Production,” Archives of Razi Institute 80, no. 5 (2025): 1103–1112. 10.32598/ARI.80.5.3442 PMC13222437 42226986

[13] R. A. El Arab , M. Alkhunaizi , Y. N. Alhashem , et al., “Artificial Intelligence in Vaccine Research and Development: An Umbrella Review,” Frontiers in Immunology 16 (2025): 1–12, 10.3389/fimmu.2025.1567116. PMC12095282 40406131

[14] V. N. R. Bandaru , S. U. Kiruthika , G. Rajasekaran , and M. Lakshmanan , “Device Aware VOD Services With Bicubic Interpolation Algorithm on Cloud,” in 2020 IEEE 4th Conference on Information & Communication Technology (CICT), Chennai, India (2020), 5–9, 10.1109/CICT51604.2020.9312118.

[15] M. Madheswaran , R. Dhanalakshmi , and G. Ramasubramanian , “Advancements in Immunization Management for Personalized Vaccine Scheduling With IoT and Machine Learning,” in The proceedings of the 10th International Conference on Communication and Signal Processing (ICCSP 2024) (2024), 1566–1570, 10.1109/ICCSP60870.2024.10543782.

[16] M. Lakshmanan , G. S. A. Mala , and G. Sivakumar , “A Secure and Efficient Framework for Storing Medical Data Using Blockchain and Cloud Servers,” in The 2024 8th International Conference on Electronics, Communication and Aerospace Technology (ICECA 2024) (2024), 1661–1667, 10.1109/ICECA63461.2024.10800855.

[17] R. Raj and S. P. Raja , “Revolutionizing Healthcare: Blockchain's Transformative Applications for Data Security, Privacy, and Interoperability,” in 2024 IEEE 9th International Conference for Convergence in Technology (I2CT) (2024), 1–9.

[18] I. Yaqoob , K. Salah , R. Jayaraman , and Y. Al‐Hammadi , “Blockchain for Healthcare Data Management: Opportunities, Challenges, and Future Recommendations,” Neural Computing and Applications 34 (2022): 11475–11490, 10.1007/s00521-020-05519-w.

[19] E. Olagunju , “Integrating AI‐Driven Demand Forecasting With Cost‐Efficiency Models in Biopharmaceutical Distribution Systems,” International Journal of Engineering Technology Research & Management 06, no. 06 (2022): 189–204.

[20] S. Sengottaiyan , S. Subbarayan , V. Viswanathan , and P. Pugazhendi , “Optimized Machine Learning With Hyperparameter Tuning and Response Surface Methodology for Predicting Tribological Performance in Bio‐Composite Materials,” Polymer Composites 45 (2024): 9421–9439, 10.1002/pc.28418.

[21] S. Saravanakumar , S. Sathiyamurthy , and V. Vinoth , “Enhancing Machining Accuracy of Banana Fiber‐Reinforced Composites With Ensemble Machine Learning,” Measurement 235 (2024): 114912, 10.1016/j.measurement.2024.114912.

[22] S. Ahamed Ali , Y. Justindhas , and M. Lakshmanan , “Secured Cloud Data Outsourcing Model Using Two Party Integrity Scheme,” in 2022 2nd International Conference on Advance Computing and Innovative Technologies in Engineering (ICACITE) (2022): 509–512, 10.1109/ICACITE53722.2022.9823507.

[23] B. Putz , F. Menges , and G. Pernul , “A Secure and Auditable Logging Infrastructure Based on a Permissioned Blockchain,” Computers & Security 87 (2019): 101602, 10.1016/j.cose.2019.101602.

[24] A. Ali , A. Khan , M. Ahmed , and G. Jeon , “BCALS: Blockchain‐Based Secure Log Management System for Cloud Computing,” Transactions on Emerging Telecommunications Technologies 33 (2022): e4272, 10.1002/ett.4272.

[25] A. Y. Cil , D. Abdurahman , and I. Cil , “Internet of Things Enabled Real Time Cold Chain Monitoring in a Container Port,” Journal of Shipping and Trade 7 (2022): 9, 10.1186/s41072-022-00110-z.

[26] S. Awasthi , P. S. Pandey , and P. C. Vashisth , “IoT‐Driven Cold Chain Management for Real‐Time Monitoring and Alerts for Perishable Goods,” 2025 International Conference on Pervasive Computational Technologies (ICPCT) (2025), 1008–1012.

[27] M. A. Naeem , X. Fang , and M. Adnan , Expert and Intelligent Vaccine Supply Chain Management for a Health Care System: A Comprehensive Survey (AuthoreaCom, 2025).

[28] S. M. H. Amiri , M. M. Islam , M. S. Hossen , et al., “Blockchain‐Enabled Solutions for Enhancing Supply Chain Transparency and Traceability,” International Journal of Science and Research Archive 16 (2025): 928–945, 10.30574/ijsra.2025.16.1.2102.

[29] S. Abdallah and N. Nizamuddin , “Blockchain‐Based Solution for Pharma Supply Chain Industry,” Computers & Industrial Engineering 177 (2023): 108997, 10.1016/j.cie.2023.108997.

[30] D. K. Rajak , B. Khanal , and M. S. A. Ansari , “Scalable Blockchain‐Based Solution for Drug Tracking in Pharmaceutical Supply Chain,” Computers & Industrial Engineering 211 (2026): 111655, 10.1016/j.cie.2025.111655.

[31] M. Lakshmanan and G. S. A. Mala , “Augmented Random Search Based Autonomous Driving System,” in 2023 5th International Conference on Smart Systems and Inventive Technology (ICSSIT) (2023), 1421–1424, 10.1109/ICSSIT55814.2023.10061010.

[32] Y. Tsang , K. Choy , C. Wu , et al., “An IoT‐Based Cargo Monitoring System for Enhancing Operational Effectiveness Under a Cold Chain Environment,” International Journal of Engineering Business Management 9 (2017): 1847979017749063, 10.1177/1847979017749063.

[33] L. Calderoni , D. Maio , and L. Tullini , “Benchmarking Cloud Providers on Serverless IoT Back‐End Infrastructures,” IEEE Internet of Things Journal 9 (2022): 15255–15269, 10.1109/JIOT.2022.3147860.

[34] W. Akram , R. Joshi , T. Haider , et al., “Blockchain Technology: A Potential Tool for the Management of Pharma Supply Chain,” Research in Social and Administrative Pharmacy 20 (2024): 156–164, 10.1016/j.sapharm.2024.02.014. 38423927

[35] S. Reno and S. Chowdhury , “Hyperledger Based Private Blockchain Technology in Pharmaceutical Manufacturing Industry for Preventing Substandard Drug Production,” 2021 International Conference on Smart Generation Computing, Communication and Networking (SMART GENCON) (2021), 1–8.

[36] M. Lakshmanan , Y. Justindhas , S. Ahamed Ali , and A. Moorthy , “Survey on Autonomous Vehicles Using Artificial Intelligence,” in 2022 3rd International Conference on Electronics and Sustainable Communication Systems (ICESC) (2022), 1237–1241, 10.1109/ICESC54411.2022.9885422.

[37] A. Gaye , B. N. Apaflo , and A. A. Donkor , “Evaluating the Effectiveness of AI‐Driven Anomaly Detection in Food Safety Compliance Monitoring,” International Journal of Frontline Research in Life Science 3 (2025): 001–009, 10.56355/ijfrls.2025.3.2.0022.

[38] M. Lakshmanan and G. S. A. Mala , “Emotional Analysis Through Deep Learning Models,” in 2022 Third International Conference on Smart Technologies in Computing, Electrical and Electronics (ICSTCEE) (2022), 1–5, 10.1109/ICSTCEE56972.2022.10100188.

